# Classifying and assembling two-dimensional X-ray laser diffraction patterns of a single particle to reconstruct the three-dimensional diffraction intensity function: resolution limit due to the quantum noise

**DOI:** 10.1107/S010876731200493X

**Published:** 2012-03-22

**Authors:** Atsushi Tokuhisa, Junichiro Taka, Hidetoshi Kono, Nobuhiro Go

**Affiliations:** a Riken Harima Institute, 1-1-1 Kouto, Sayo-cho, Sayo-gun, Hyogo, 679-5148, Japan; b Quantum Beam Science Directorate, Japan Atomic Energy Agency, 8-1-7 Umemidai, Kidugawa-shi, Kyoto, 619-0215, Japan; c XFEL Division, Japan Synchrotron Radiation Research Institute, 1-1-1 Kouto, Sayo-cho, Sayo-gun, Hyogo, 679-5198, Japan

**Keywords:** biological macromolecules, classification of two-dimensional diffraction patterns, common intersecting circles, attainable structural resolution

## Abstract

A new two-step algorithm is developed for reconstructing the three-dimensional diffraction intensity of a globular biological macromolecule from many experimentally measured quantum-noise-limited two-dimensional X-ray laser diffraction patterns, each for an unknown orientation. The first step is classification of the two-dimensional patterns into groups according to the similarity of direction of the incident X-rays with respect to the molecule and an averaging within each group to reduce the noise. The second step is detection of common intersecting circles between the signal-enhanced two-dimensional patterns to identify their mutual location in the three-dimensional wavenumber space. The newly developed algorithm enables one to detect a signal for classification in noisy experimental photon-count data with as low as ∼0.1 photons per effective pixel. The wavenumber of such a limiting pixel determines the attainable structural resolution. From this fact, the resolution limit due to the quantum noise attainable by this new method of analysis as well as two important experimental parameters, the number of two-dimensional patterns to be measured (the load for the detector) and the number of pairs of two-dimensional patterns to be analysed (the load for the computer), are derived as a function of the incident X-ray intensity and quantities characterizing the target molecule.

## Abstract 2

A new algorithm is developed for reconstructing the high-resolution three-dimensional diffraction intensity function of a globular biological macromolecule from many quantum-noise-limited two-dimensional X-ray laser diffraction patterns, each for an unknown orientation. The structural resolution is expressed as a function of the incident X-ray intensity and quantities characterizing the target molecule.

## 1. Introduction

New, intense X-ray free-electron laser (XFEL) light sources offer a new possibility in imaging single biological macromolecules. The main problems to be solved for realization of this possibility originate from the extreme weakness of the scattered light from a single molecule. One problem is severe damage of a target caused by a single shot of intense X-ray light used to compensate for the weakness. In this respect, a lower intensity of incident X-rays is preferred. Another problem due to the weakness is the quantum noise. Algorithms for structure determination must be developed to process the experimental data immersed in the quantum noise. From this perspective, a higher intensity of incident X-rays is preferred. This paper focuses attention on this latter problem. For this purpose we make a tentative assumption that the damage process can be neglected, and will clarify the mechanism of how the quantum noise sets a limit on the resolution of structure determination. In other words, we are interested in this paper only in the lower bound of the incident X-ray intensity.

The damage problem prevents the possibility of using the same molecule repeatedly as a target. Instead we assume that a target macromolecule assumes a well defined three-dimensional structure and a new molecule from an ensemble of the same molecules is placed repeatedly at the target position, but unfortunately in an unknown random orientation. A macromolecular complex with a definite molecular composition and three-dimensional structure can also be treated. The term ‘molecule’ is used to mean both a biological macromolecule and its complex. Extension of the results of this paper to cases of large-scale conformational fluctuations with a magnitude larger than the resolution of structure determination will be addressed in a future paper. Because of this limitation, we specifically exclude fibrous macromolecules and assume that molecules are globular with a more-or-less spherical shape. Note also that we develop analyses in this paper under idealizing (as compared with probable experimental realizations) assumptions about the state of the target molecule such as (i) ideally random orientations and (ii) the absence of hydrating water molecules. Adaptation to these realistic problems will also be treated in future papers.

A measurable two-dimensional diffraction intensity pattern depends on an unknown molecular orientation. This missing orientational information is to be recovered computationally during an analysis of a set of many two-dimensional intensity patterns. Also missing in a two-dimensional intensity pattern is the phase information necessary for derivation of a three-dimensional molecular structure. This missing phase information is also to be recovered computationally by the so-called oversampling method (Fienup, 1982 ▶; Elser, 2003 ▶).

The methods of single-particle imaging by XFEL can be classified into two paths, depending on which of the two types of missing information is recovered first. In the first, path *A*, method, a computational procedure is applied to a set of phase-missing two-dimensional intensity patterns to find their mutual locations in the three-dimensional wavenumber space. When a sufficient number of two-dimensional patterns are properly located, a three-dimensional diffraction intensity function can be constructed, to which the oversampling method is applied to recover the missing phase information. Together with this phase information, a three-dimensional real-space structure can be derived by an inverse Fourier transformation. In the second, path *B*, method, the oversampling method is applied to each of the measured two-dimensional intensity patterns. Together with the recovered phase information, a two-dimensional real-space structure is obtained by an inverse Fourier transformation, which is approximately a projection of a three-dimensional real-space structure along an axis of the incident X-ray beam. Such two-dimensional structures of a minivirus particle as revealed by a single-shot 6.9 Å hard-X-ray free-electron laser have been recently reported (Seibert *et al.*, 2011 ▶). From many projected two-dimensional images thus obtained, a three-dimensional real-space structure can be constructed by applying the method of tomography. Such a three-dimensional human chromosome structure as revealed by coherent 2.5 Å X-rays from synchrotron radiation has been reported (Nishino *et al.*, 2009 ▶).

Because of the weakness of scattered light from a single molecule, the quantum noise is a serious problem especially at high-angle pixels. The quantum noise appears to limit the resolution of a three-dimensional real-space structure to be obtained at the end, though by different mechanisms in paths *A* and *B*. In path *A*, the quantum noise is expected to set a resolution limit to locating a two-dimensional intensity pattern in the three-dimensional wavenumber space. Because photon-count data at many pixels can be considered integrally in a computational procedure to find a location, effective information seems extractable even from high-noise data at pixels with an expected mean photon count smaller than unity. Even though data at high-angle pixels are very noisy in each of the two-dimensional intensity patterns thus located, data at similar locations can be averaged to reduce the noise. The attainable structural resolution is determined by the wavenumber of a limiting pixel, from the data of which effective information can be extracted. In path *B*, where the oversampling method is applied directly to each two-dimensional intensity pattern, the quantum noise is expected to set a limit on the applicability of this method. When high-noise data from high-angle pixels with an expected photon count smaller than unity are included, the phase recovery procedure is expected to fail to converge and to cease to work. Therefore a pixel with an expected photon count of unity appears to be a limiting pixel, and its wavenumber appears to give the resolution. From such an analysis we expect that the path *A* analysis is better in extracting effective information from noisy data and therefore in deriving higher-resolution real-space structures. For this reason we are interested in this paper in developing a path *A* method. Of course, superior situations of the path *B* method are conceivable depending on problems of developing detecting devices and sample preparation, and also on the biological significance of the results obtained.

Methods hitherto proposed to find the locations of individual two-dimensional intensity patterns in the three-dimensional wavenumber space in path *A* methodology can be classified into two groups. In group 1 (Huldt *et al.*, 2003 ▶; Bortel & Faigel, 2007 ▶; Shneerson *et al.*, 2008 ▶; Bortel *et al.*, 2009 ▶; Yang *et al.*, 2010 ▶), a method of finding similarity between an arbitrary pair of two-dimensional intensity patterns is prepared. Then, a set of two-dimensional patterns are classified into groups of similar patterns according to this similarity, which are then averaged to reduce the quantum noise. Then, for an arbitrary pair of noise-reduced intensity patterns, their mutual location in the three-dimensional wavenumber space is identified by finding an intersecting circle between them. A three-dimensional diffraction intensity function can be constructed when a sufficient number of two-dimensional patterns are properly located in the three-dimensional wavenumber space. In the methods of group 2 (Fung *et al.*, 2009 ▶; Loh & Elser, 2009 ▶; Elser, 2009 ▶; Loh *et al.*, 2010 ▶), a tentative three-dimensional diffraction intensity function (or a function of similar mathematical setting) is assumed. Then, each two-dimensional intensity pattern is located so as to best fit in this three-dimensional intensity function. From a set of two-dimensional patterns thus located, the three-dimensional diffraction intensity function is updated. By repeating this cycle of best fitting and updating, an ultimate three-dimensional diffraction intensity function is obtained. Even though the methods of the second group appear promising, the demonstrated abilities of the individual methods proposed so far are limited.

We focus attention in this paper on developing an algorithm beyond existing methods of single-particle imaging belonging to the path *A*, group 1 methodology. New developments have been attained in two aspects. First, we will develop and improve methods of computational analyses and procedures to arrange a set of many experimentally measurable two-dimensional intensity patterns in the three-dimensional wavenumber space so that a three-dimensional intensity function can be constructed. The newly developed method enables us to attain higher-resolution structures. Second, we will derive explicit theoretical expressions for two main parameters which govern the number 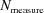 of two-dimensional patterns to be measured (the load for the measuring machine) and the number 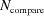 of pairs of two-dimensional patterns to be compared (the main computational load for analyses), as well as for the space resolution attainable from the analysis of the data, in terms of (*a*) the X-ray intensity used for the measurement and two types of quantities characterizing a target; (*b*) the Shannon molecular length (or, simply, molecular length) 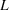 (the length of a side of the smallest cubic box that can contain a target globular molecule); and (*c*) the radial diffraction intensity density function 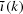 (the average of the the squared modulus of the structure-factor function on a sphere 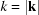 in the wavenumber space). The achievement of the second aspect provides basic information for designing new experiments and experimental instruments.

The results in the two aspects are obtained in this paper by taking advantage of simulated diffraction intensity data for a protein, lysozyme, and a protein complex, HslUV complex, for which structural atomic coordinates are available from the Protein Data Bank (PDB) (Berman *et al.*, 2000 ▶). The former (Weaver & Matthews, 1987 ▶) (PDB code 2lzm, number of residues 164, molecular length 60 Å) is chosen as a typical small globular protein. The latter (Sousa *et al.*, 2002 ▶) (PDB code 1kyi, total number of residues 7416, molecular length 200 Å) is chosen from very large protein complexes in the PDB with more-or-less globular shape. But it is in a sense an atypical complex, because it has a big hollow space inside the structure. Simulations are carried out by assuming the wavelength of incident X-rays 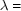 1 Å and for various intensities. The Shannon molecular length 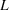 and radial diffraction intensity density function 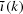 are determined for these two targets from their respective PDB atomic coordinates, and are used to estimate numerical values of the two main experimental parameters, 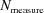 and 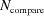, and also of the attainable resolution as functions of the incident X-ray intensity.

To make the results of this paper more useful for designing new experiments and experimental instruments, the theory must be extended so as to be applicable even for structure-unknown molecules. This objective is studied in a separate paper.

This paper is ordered as follows. In §2, we will discuss a method of finding similarity between an arbitrary pair of two-dimensional diffraction intensity patterns. This method is used to classify two-dimensional patterns into groups of similar patterns. In §3, we will treat the problem of finding relative orientations between groups of similar patterns, which is information to be used for assembling two-dimensional intensity patterns into a three-dimensional diffraction intensity density function. In §4, we will give a somewhat detailed summary. Readers may find it easier to comprehend §§2 and 3 by reading §4 in parallel. Appendices *A* [App appa], *B* [App appb] and *C* [App appc] describe mathematical derivations of relations used in §§2 and 3.

## 2. Classification of two-dimensional diffraction intensity patterns

### 2.1. Two-dimensional diffraction pattern on an Ewald sphere

Here we define notations of pertinent quantities. The experimentally observable diffraction intensity 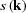, given in the unit of a number of photons arriving at a pixel of the detector of solid angle 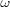, is given, except for a phase factor, by
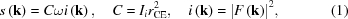
where 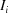 is the incident X-ray intensity (given, in the following, in the unit of a number of photons per pulse of free-electron laser per mm^2^), 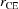 is the classical electron radius, 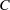 is a coefficient given by these quantities, 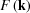 is the structure factor, 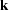 is the momentum transfer and 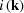 is the diffraction intensity density. The magnitude of the momentum transfer is given by 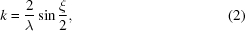
where 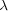 is the X-ray wavelength and 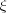 is the angle of diffraction. Note that, even though this angle is expressed as 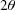 in the usual literature, we nevertheless express it as 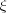 because this quantity, having a meaning as part of a certain polar angle, has an important role in this paper. Even though the structure factor 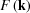 is a continuous function in the wavenumber space, its squared modulus is measured experimentally by a detector with an array of finite-sized pixels. When 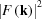 is discretely sampled at lattice points of a cubic lattice with a lattice constant of 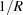, it corresponds to the use of the detector pixel size of 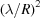 in solid angle, *i.e.*

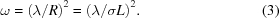
In this expression, 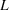 is the length of a side of the smallest cubic box (Shannon box) that can contain a target globular molecule. We shall refer to this quantity as the Shannon molecular length or simply the molecular length. From the point of view of the oversampling method for phase retrieval (Fienup, 1982 ▶; Elser, 2003 ▶), the detector pixel size must be chosen so that 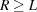. The ratio 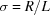 is called the linear oversampling ratio. A pixel with 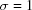 will be referred to as a Shannon pixel.

The quantity of equation (1) is an expected number of photons arriving at a detector pixel. However, in real experiments, what is measured is an integral number of photons, given by the quantum-mechanical probability. In this paper we simulate this probability by replacing the function 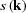 of equation (1) with a stochastic function 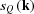 which assumes only integral values according to the Poisson distribution. To distinguish these two functions, let us call the quantity of equation (1) the theoretical diffraction intensity, and the replaced stochastic function the experimental diffraction intensity.

**Figure 1:**
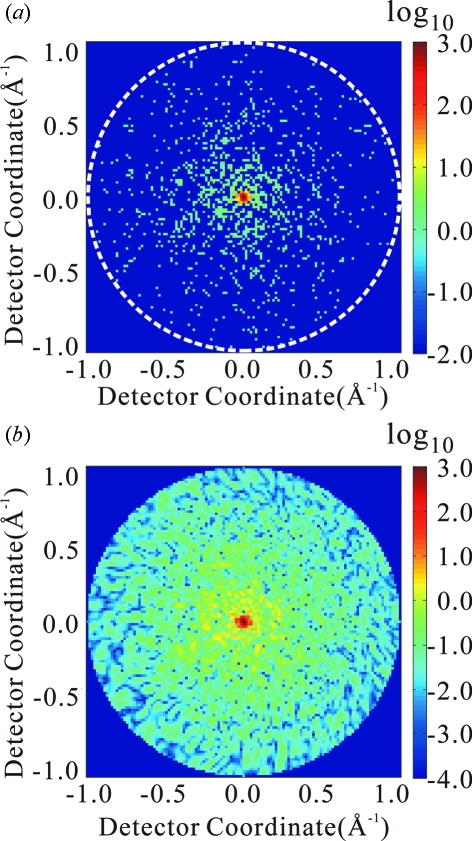
Examples of two-dimensional diffraction intensity patterns simulated for lysozyme by assuming the wavelength of the incident X-rays 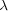 = 1 Å and the intensity 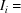 5 × 10^21^ photons pulse^−1^ mm^−2^. In (*b*), the theoretically expected mean number of diffracted photons arriving at a Shannon pixel is shown. In (*a*), the number of photons is an integer, chosen according to the Poisson distribution having the theoretically expected mean value as its mean. For this assumed intensity, the mean count of 0.1 photon is observed at 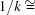 2 Å. In this figure both the abscissa and ordinate show detector coordinates in the sense that they are proportional to coordinates on a flat detector on which the surface of an Ewald sphere is radially projected from its centre (central azimuthal projection).

Examples of a simulated two-dimensional experimental and a theoretical diffraction intensity pattern are shown in Figs. 1 ▶(*a*) and 1 ▶(*b*), respectively, for the case of lysozyme. We see that, when theoretical diffraction intensities are much less than unity, experimental diffraction intensity values at most pixels vanish. From such noisy data, we have to guess the correct mean values. These are patterns for which we develop a method of analysis for structure determination. For this purpose we need some theoretical tools. A mathematical expression of a two-dimensional pattern for a molecule with a given orientation is explored in detail in Appendix *A* [App appa]. A molecular orientation is described by a Eulerian angle 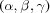 with its corresponding 3 × 3 orthogonal matrix 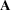 given by equation (27). The Eulerian angle is defined so that, out of a set of three angles, 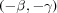 are polar angles of the direction of the incident X-ray beam with respect to the molecule, and 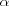 is an angle of rotation of the detector plane around the axis of the incident beam. A two-dimensional pattern is given by the quantity of equation (1) on the surface of an Ewald sphere. By introducing a polar coordinate 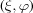 on the surface of an Ewald sphere, the two-dimensional pattern is given from equations (40), (39), (35) and (42) by 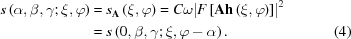
This equation means that the Ewald spheres corresponding to 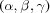 and 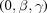 are essentially the same spheres giving the same surface.

### 2.2. High correlation line in a correlation pattern

As outlined in §1 we adopt in this paper the basic strategy in which, after measurement of a large number of two-dimensional patterns for a molecule in unknown random orientations, we classify them into groups of similar patterns. In the following we consider a pair of Eulerian angles 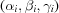 and 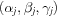 with corresponding matrices 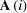 and 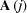, and two-dimensional patterns 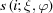 and 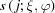. For this pair we will be interested in an angle 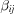 between the two beam directions 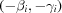 and 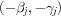, which satisfies 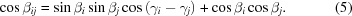
This angle plays the role of a measure of similarity between a pair of two-dimensional patterns. When it is very small, we classify them into one group of similar patterns even for very different 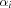 and 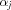.

Our problem is to judge, for a given pair of experimental two-dimensional patterns such as those shown in Fig. 1 ▶(*a*), whether or not they are realizations of a similar theoretical two-dimensional pattern. The starting point is the calculation of a correlation function as was originally proposed by Huldt *et al.* (2003 ▶). In this treatment we are interested in pixels on a circle with a fixed value of 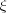. Let the number of pixels on a circle be 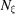. Bortel & Faigel (2007 ▶) proposed to pre-normalize the data on a circle to have uniform second moments for the calculation of a correlation function. More recently, they (Bortel *et al.*, 2009 ▶) proposed further to pre-normalize so as to have vanishing mean and uniform second moment and also to compare axially rotated patterns. In our method we normalize the data on a circle to have a uniform mean and are interested in the correlation of their deviation from the mean after they are mutually rotated by an angle 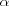. This means that we are concerned with the following correlation function: 
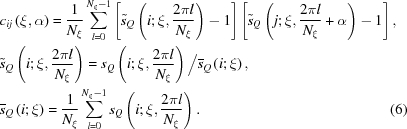
This quantity can also be expressed as follows: 
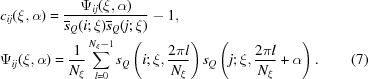
The correlation function of equation (6) is calculated as a two-dimensional function of 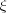 and 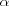, which will be referred to as a correlation pattern. The merits of using the normalized quantity of equation (6) are to normalize for variations of intensities over different circles and for experimental variations of the pulse intensities of XFELs.

**Figure 2:**
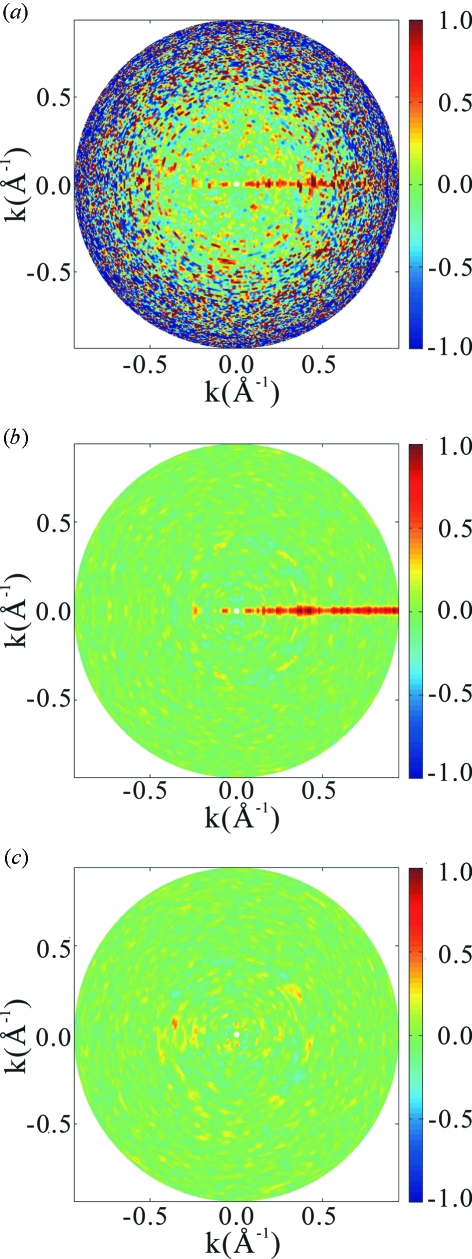
Correlation pattern for lysozyme by assuming the intensity of the incident X-rays 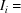 5 × 10^21^ photons pulse^−1^ mm^−2^. (*a*) Correlation pattern of equation (6) for a pair of two-dimensional experimental intensity patterns obtained from an identical two-dimensional theoretical intensity pattern but with different sets of random numbers for the Poisson distribution. A *high correlation line* is observed extending from the origin towards the direction of 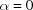. The high correlation line fades at a certain value of 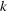 because of the quantum noise. (*b*) Correlation pattern of equation (8), which is obtained by averaging the pattern shown in (*a*) over the quantum noise. Because of the suppressed quantum noise, the high correlation line is now observed extending to high 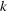 values. (*c*) When the directions of the beam axes are significantly different in a pair of two-dimensional intensity patterns, the high correlation line is absent. In this and subsequent figures (Figs. 2 ▶, 4 ▶ and 8 ▶), unlike in Fig. 1 ▶, both the abscissa and ordinate are taken to be proportional to 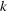. In this case, the area in the figures is also proportional to that on the curved Ewald spheres (Lambert’s azimuthal equal-area projection).

An example of a correlation pattern is shown in Fig. 2 ▶(*a*). In this figure the pattern is shown not as a function of 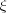 and 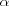 but as a function of 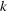 and 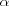, where 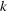 is given by equation (2). In a general case where the angle 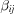 between the two beam directions is not very small, this correlation pattern appears to be a random function of 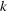 and 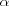. Let us write such 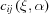 as 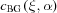, where BG stands for background. When 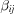 is very small, there appears *a line of high correlation* for a certain value of 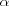. In the particular case of Fig. 2 ▶(*a*), the two beam directions are identical, and therefore both 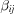 and 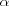 vanish.

The correlation pattern of equation (6) is heavily affected by the quantum noise. When the quantum noise is suppressed, this quantity is given approximately by 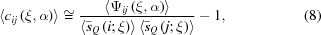
where 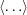 is a mean averaged over the quantum noise. To derive this expression we introduced an approximation of taking the means of three factors independently, an approximation which is good when standard deviations of the three factors are significantly smaller than their respective means. Examples of this quantity are shown in Figs. 2 ▶(*b*) and 2 ▶(*c*). Fig. 2 ▶(*c*) shows a pair of diffraction patterns for which the value of 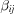 is not small. This is an example of 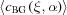. Even though Figs. 2 ▶(*b*) and 2 ▶(*c*) contain no effect of quantum noise, the pattern other than the high correlation line appears to be rather random. Such a behaviour should be a consequence of an appearance of the diffraction intensity density function 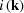 that can be captured as a stochastic function.

**Figure 3:**
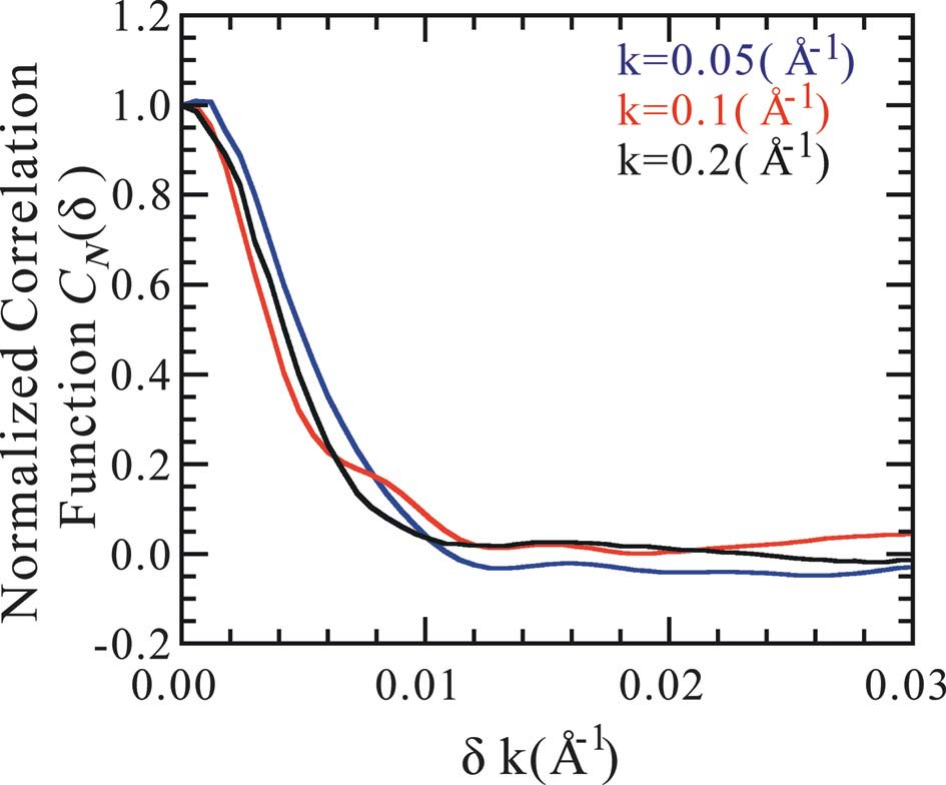
Normalized correlation function 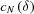 of equation (9) for the space correlation of the values of 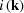 on a sphere 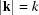 of radius 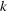 for the HslUV complex. The angle 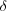 is shown in the abscissa as a product with 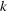. For 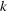 equal to or larger than 0.2 Å^−1^, it is given to a very high accuracy by a Gaussian function. Data only up to the case of 0.2 Å^−1^ are shown in the figure. As 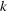 becomes smaller, slight deviations from the Gaussian behaviour are observed.

To characterize the diffraction intensity density function from such a point of view, we studied first how values of 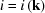 are distributed on a sphere of 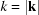 around its mean 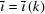. For this purpose, 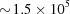 points are sampled randomly with a uniform probability on each sphere, and the value of 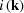 is calculated at each sampled point. We confirmed that, except for small values of 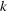, the distribution is given to a very good accuracy by the exponential distribution as was originally discovered by Wilson (1949 ▶). Moreover, as shown in Fig. 3 ▶, it is found empirically that, except for small values of 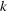, values of 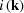 on the sphere of 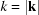 are correlated in such a way as to satisfy the following simple relation, 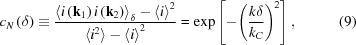
where 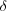 is an angle between the two 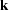 vectors, the average is taken over all pairs of vectors with a given value 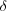, and the correlation length 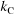 has been found to be independent of the value of 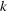 and is given approximately by 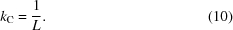
Because, as shown by Wilson (1949 ▶), the exponential distribution is a consequence of the irregular three-dimensional structures of biopolymers at the atomic level, we shall refer to the empirically observed distribution as a *structure irregularity distribution*.

To derive the mean behaviour of 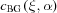, we average 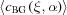 over the structure irregularity distribution. As shown in Appendix *C* [App appc], we see that 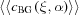 vanishes. This is reasonable because when there is no correlation between two points on two circles appearing in equation (6) an average can be taken on each of 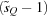 to yield a vanishing result.

**Figure 4:**
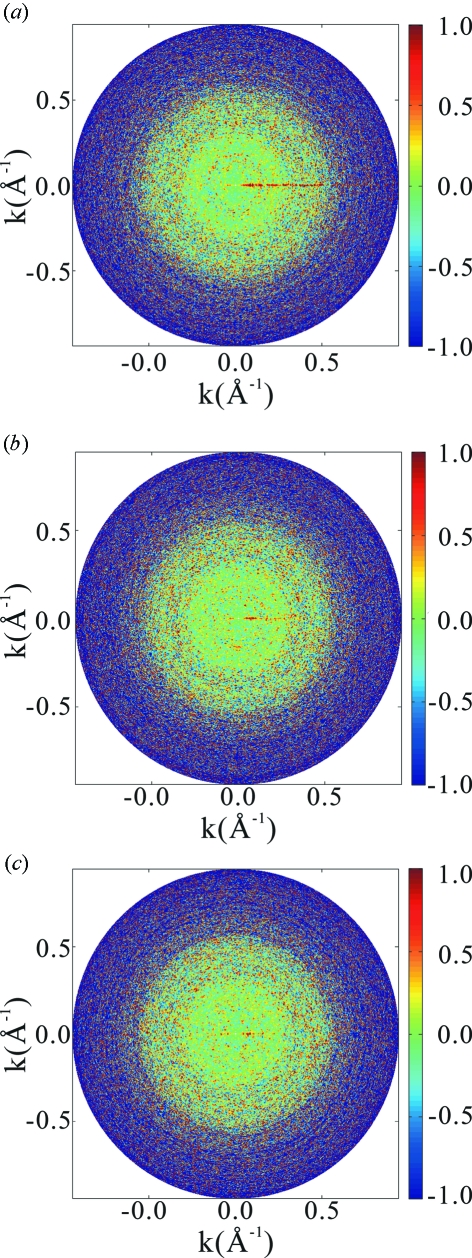
Correlation patterns for the HslUV complex and by assuming the intensity of incident X-rays 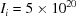
 photons pulse^−1^ mm^−2^. (*a*), (*b*) and (*c*) are for the angle 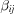 between the two beam directions being 0, 1 and 3°, respectively. Pixels having a value larger than 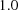 are shown by the colour code of 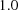. We see in this figure that, for 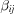 = 3°, the high correlation line is barely visible.

Further examples of a correlation pattern of equation (6) are shown in Fig. 4 ▶. Because the mean of the correlation pattern vanishes except for a high correlation line, a mathematical expression of the high correlation line should be obtained as a mean of the correlation pattern over the two distributions, the Poisson distribution and the structure irregularity distribution. As shown in Appendix *C* [App appc] it is given to a good approximation by the following expression: 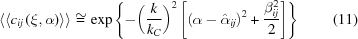
where 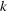 is a function of 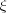 through equation (2) and the direction of the high correlation line, 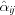, is given to the zeroth order of the small quantity 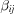 by 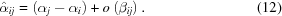
It should be noted that 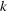 appears in equation (11) as a quantity normalized by 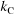, or, because of equation (10), as a product 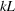.

As mentioned earlier, Bortel *et al.* (2009 ▶) proposed to use a quantity pre-normalized to have a vanishing mean and uniform second moment. However, when we apply our analysis to their proposed correlation pattern, an expression similar to equation (11) is obtained but with an additional factor which is approximately 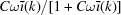 and becomes smaller for larger 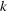. Because of this additional factor, the quantity they employed for detecting similarity between two-dimensional patterns is less sensitive for high 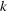 values. This explains why our method is more sensitive for higher 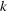 values.

### 2.3. Identifying the high correlation line against the noisy background and attainable resolution

**Figure 5:**
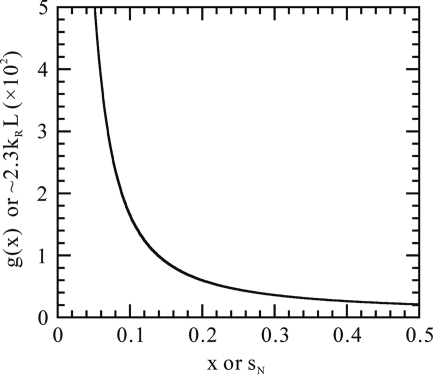
Graph of the function of equation (14). The abscissa 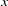 and ordinate 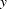 mean physically an expected number 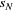 of photons arriving at a Shannon pixel at 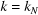 and 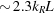, respectively.

**Figure 6:**
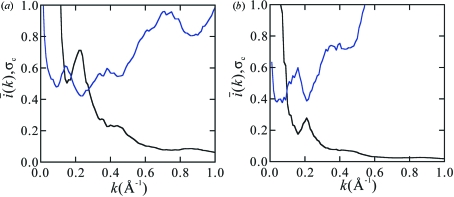
Plot of 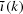, the average of 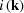 of equation (1) on the sphere of 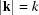 (black line), and 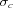 of equation (13) (blue line) for lysozyme (*a*) and the HslUV complex (*b*). The intensities assumed are 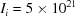 and 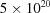
 photons pulse^−1^ mm^−2^, respectively.

In Fig. 4 ▶ we see that, even though the mean value of the correlation pattern should vanish in the background, its actual values become very noisy for larger values of 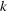. This is due to both the quantum noise and the structure irregularity distribution. Identification of the high correlation line would be affected by the noise. (Note that we are here treating the structure irregularity distribution as a part of the noise.) The level of noise in the quantity of equation (6) can be expressed by its standard deviation. As derived in Appendix *C* [App appc], it is given by 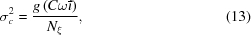
where the function 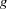 is defined by 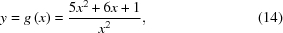
and 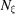 is given by 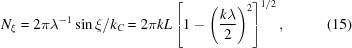
which means that we are assuming Shannon pixels. Fig. 5 ▶ shows the graph of 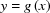. Fig. 6 ▶ is a plot of the standard deviation given by equation (13), which is a globally increasing function of 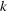 in the high-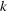 region. When averaged over the two distributions, the Poisson distribution and the structure irregularity distribution, the peak value of the high correlation line for a given value of 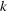 (or, equivalently 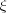) is 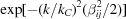 according to equation (11), which is a decreasing function of 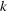. It is expected that the actual high correlation line is observable roughly up to 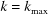, where the mean peak value becomes equal to the standard deviation 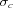, *i.e.*

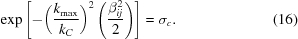
In fact, in Figs. 2 ▶(*a*) and 4 ▶(*a*) for cases of 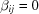 and therefore where the mean peak value should stay unity, the actual high correlation lines are observed for lysozyme up to 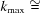 0.7 Å^−1^ and for the HslUV complex up to 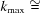 0.55 Å^−1^, where 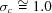 according to Fig. 6 ▶. In Fig. 4 ▶(*b*) for the case of the HslUV complex with 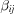 =  1°, the actual high correlation line is observable up to 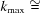 0.3 Å^−1^, where 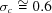 according to Fig. 6 ▶ and equation (16) is roughly satisfied.

From equation (16) and Fig. 6 ▶, we see that we can derive the value of 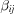 from the measured length 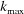 of the high correlation line. The longer the length 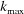, the smaller the value of 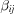. From the value of 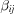, we judge the similarity of a pair of two-dimensional patterns. When judged similar, they are classified into the same group. After the classification, two-dimensional patterns classified into the same group are averaged in order to improve the signal-to-noise ratio. Because this averaging is done for patterns with slightly different directions of the incident beam, the resolution of the resulting three-dimensional structure will be affected. In order to attain the highest possible resolution, we should adopt a strategy in which we classify a pair of two-dimensional patterns into the same group when their high correlation line reaches the highest possible *k* region. Let us define 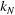 (subscript 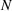 for noise) as the lower bound of such a region. This quantity 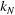, *the limiting* 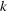 *value* for correlation recognition, plays a central role in the method of single-particle imaging developed in this paper. In the case of Fig. 4 ▶(*a*) we judge that the high correlation line extends up to such a region, where the line can no longer be distinguished from the background. In the case of Fig. 4 ▶(*b*) the line appears to have faded away before reaching such a region. The limiting value 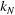, to be determined purely operationally in real applications, appears more-or-less well defined. However, we need to interpret the value of 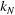 in a more theoretical setting. Because it defines the lower bound of the noisy region, it should be characterized by its value of 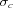. From Figs. 4 ▶(*a*) and 4 ▶(*b*) we see that it should be between 0.6 and 1.0. As a modest estimate, we assume that 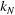 corresponds to the value of 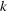 at which 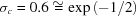. Then, from equation (16) we see that the corresponding value of 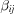 is estimated to be within 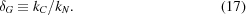



Let us now assume that a classification group of similar two-dimensional patterns is constructed by a group of two-dimensional patterns with 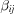 within this angle from a certain reference two-dimensional pattern. Note that the average distance (root-mean-square distance) between a pair of two-dimensional patterns in this classification group is also given by 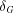. During the procedure of averaging, two-dimensional patterns rotated by 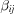 around the origin are averaged. The magnitude of displacement in 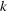 space by this rotation is given by 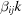, with its maximum value being 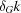. When this magnitude is smaller than the correlation length 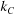 of the diffraction intensity density, the averaging procedure works to attenuate the effect of the noise. When the product becomes larger than 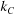, the averaging procedure works to destroy the information in two-dimensional patterns. This means that the structural information is contained in 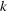 only up to 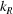 satisfying 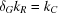. Then, from equation (17), we see 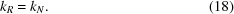

*A limiting photon count* 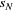, which is an expected number of photons arriving at *a limiting pixel*, a Shannon pixel at the limiting 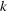 value, 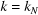, is given by 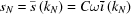. In equation (13), 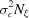 at 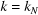 is given by 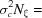 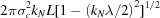. This expression can be approximated as 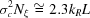, because 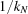 is in most cases at least a few times larger than 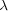. In Fig. 5 ▶ for a graph of 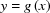, 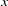 and 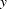 can also be interpreted as 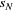 and 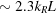, respectively. Note that *the normalized resolution*, 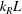, is the number of independent structural descriptive elements along the molecular length 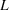. For a method of single-molecule imaging to be useful, this number should be at least 20, hopefully 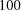. Note that this number is determined mainly by the limiting photon count 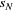. Fig. 5 ▶ shows this dependence. We see that, to attain 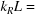 20–100, 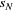 should be in the range of 0.25–0.08. We have to measure and analyse such low-photon-number data. Also this number highlights a high sensitivity of the proposed method of analysis to extracting information from noisy data.

**Figure 7:**
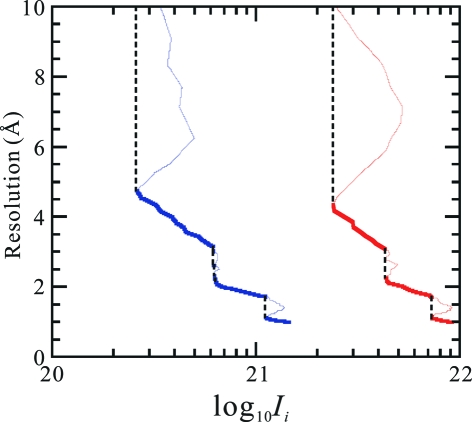
Attainable resolution as a function of the incident X-ray intensity 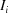 (photons pulse^−1^ mm^−2^) for lysozyme (dotted and solid right-hand lines) and the HslUV complex (dotted and solid left-hand lines). When there is more than one value of resolution for a given value of 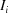, the best value indicated by a solid line can be obtained.

In the above relation between the limiting photon count 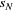 and the normalized resolution 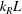, the incident X-ray intensity 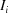 is treated as an implicit variable parameter. To identify a particular value of 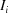 to attain a resolution 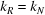, we remember the relation 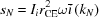 [equation (1)]. Then, by defining a function inverse to the function 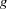 of equation (14) as 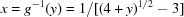, equation (13) can be transformed to 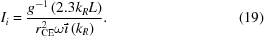
Fig. 7 ▶ shows the resolution 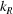 as a function of intensity 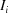 for lysozyme and the HslUV complex obtained by using this equation. When there is more than one value of resolution for a given value of 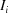, the best value can be obtained. (The high correlation line may become visible again in a high-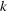 but low-noise region after once becoming invisible in a low-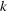 but high-noise region.)

Since the solid angle of the range of one classification group is given by 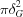, and the total solid angle of the direction of the incident beam is 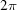 because of the centrosymmetric property of the three-dimensional diffraction intensity function, the number of classification groups 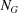 is given by
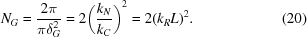



## 3. Placing two-dimensional patterns in the three-dimensional wavenumber space: a method of finding the relative orientation between two-dimensional patterns

After two-dimensional patterns are classified by the method described in the previous section, patterns classified into the same group are averaged to reduce the noise. When signal-enhanced patterns are obtained, they are to be placed in the three-dimensional wavenumber space by finding their relative orientations. The two-dimensional patterns exist on Ewald spheres. Because all these Ewald spheres have the same radii and their surfaces contain the origin 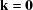 of the wavenumber space, any pair of Ewald spheres either contact at the origin or have a circular intersection, which also contains the origin. Shneerson *et al.* (2008 ▶) studied the problem of placing two-dimensional patterns in the three-dimensional space by paying attention only to an approximately straight portion of the intersecting circles near the origin 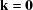. Yang *et al.* (2010 ▶) refined the method of finding the tangential direction of the intersecting circle at the origin 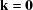 by explicitly paying attention to the curvature of the intersection. However, for the placement problem they used the method of Singer & Shkolnisky (2011 ▶) for cryo-electron microscopy in which only information on tangential directions is used. However, it is obvious that a relative orientation between a pair of Ewald spheres can be determined once their common circle is identified. In this section we first develop a method of identifying an intersecting circle for a given pair of signal-enhanced two-dimensional patterns 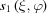 and 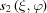, and derive a mathematical expression for the relative orientation. Second, we ask what is the necessary number of patterns to be averaged for possible identification of common circles? The actual construction of a single three-dimensional diffraction intensity function from the data of relative orientations will be treated in a different paper.

We assume that a pair of signal-enhanced two-dimensional patterns 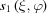 and 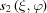 exist on Ewald spheres of as yet unknown orientations, 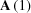 and 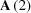. Because of the centrosymmetric property of the three-dimensional diffraction intensity function, any Ewald sphere 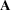 has its centrosymmetric image characterized by 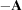. Therefore, Ewald sphere 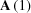 should have an intersecting circle with each of the Ewald spheres 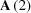 and 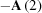. This means that for any pair of two-dimensional patterns, 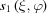 and 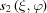, there exist two common circles. A method of finding them is developed in Appendix *B* [App appb] and here we describe only the result. Each of the two common circles exists as a circle of vanishing values in each of two groups of plots, (*a*) 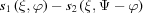 and (*b*) 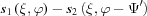, where the parameters 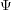 and 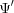 generate each of the two groups, respectively. When the plot (*a*) vanishes on a circle with its centre at 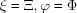 for a certain parameter value 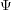, the polar coordinates of the centres of intersecting circles are 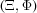 on 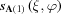 and 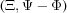 on 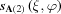. When the plot (*b*) vanishes on a circle with its centre at 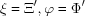 for a certain parameter value 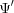, the polar coordinates of the centres of intersecting circles are 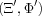 on 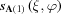 and 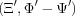 on 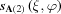. Then, the Euler angle of the relative orientation 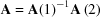 is given by 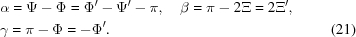
Thus, the same set of Eulerian angles 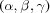 is now determined from each of the intersecting circles between Ewald spheres 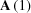 and 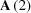, and between Ewald spheres 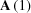 and 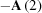. Even though the result is redundant, the actual procedures of finding vanishing circles on plots (*a*) and (*b*) are much influenced by experimental noise. In this situation, finding the same quantity simultaneously by the two methods is a desirable numerical procedure.

**Figure 8:**
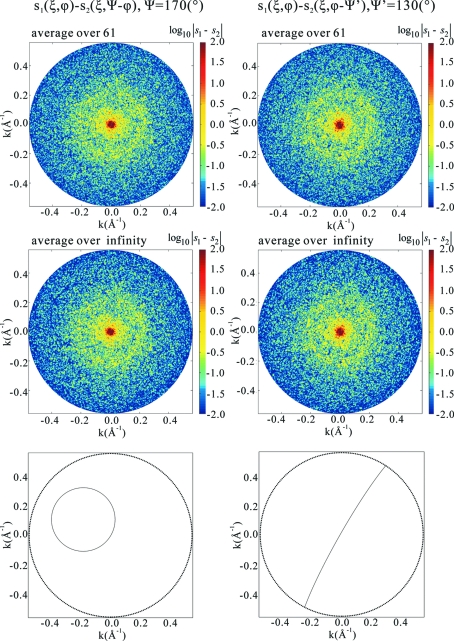
Plots for detecting an intersecting circle between a pair of two-dimensional patterns for the case of the HslUV complex and for the incident X-ray intensity 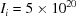
 photons pulse^−1^ mm^−2^. By careful examination of the plot, we see that intersecting circles become visible when averaging of 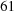 or more patterns is done in this case. In the bottom panels the intersecting circles are highlighted.

Fig. 8 ▶ shows an example of how circles of vanishing values become visible as the number of averaging patterns is increased. In the case of this example for the HslUV complex, in which the limiting photon count 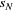 is 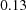, we see by inspection that averaging over about 61 patterns is necessary for the identification. This process has been done for lysozyme and the HslUV complex both for a series of values of the incident X-ray intensity. It has been found that a product of the number 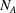 of necessary patterns and the limiting photon count 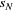 (therefore, the intensity 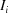 of the incident X-ray) is constant in either ‘molecule’. The analysis described in Appendix *C* [App appc] indicates that the product 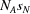 must be 8 or larger for common circles to be identified. This is exactly the number observed in the case of Fig. 8 ▶. Therefore, the necessary number of patterns is given by 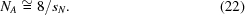
Table 1 ▶ summarizes the results obtained as applied to the two ‘molecules’.

## 4. Summary and conclusion

Two aspects of a method of single-particle imaging belonging to the path *A*, group 1 methodology have been developed. First, a new, improved method has been developed for computational analyses and procedures to arrange a set of many experimentally measurable two-dimensional diffraction intensity patterns in the three-dimensional wavenumber space. Second, explicit theoretical expressions have been derived for important experimental parameters in terms of the incident X-ray intensity and two types of quantities characterizing a target.

The number 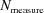 of two-dimensional patterns to be measured is given by the product 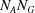 of the number of classification groups 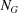 and the average number of two-dimensional patterns 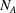 to be averaged in each group for noise reduction. The number 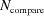 of pairs of two-dimensional patterns to be analysed is given by 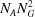, because the detection of similarity of patterns is to be carried out for each of the pairs, one from patterns representing each group and the other from all measured patterns. We derived theoretical expressions for the two parameters, 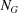 and 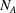.

Concerning the first aspect, we have improved a hitherto proposed method for judging whether or not an arbitrary pair of two-dimensional patterns are similar enough to belong to the same classification group. Also, we developed methods of finding common intersecting circles between an arbitrary pair of noise-reduced two-dimensional patterns, and thereby relatively locating them in the three-dimensional wavenumber space. After locating many two-dimensional diffraction patterns properly in the wavenumber space, we have to construct a single three-dimensional diffraction intensity function. This problem, as well as the problem of application of the phase retrieval procedure to such a three-dimensional function, will be treated in a different paper.

The judgment of similarity is based on a two-dimensional correlation pattern for each pair of two-dimensional diffraction intensity patterns. For the calculation of correlation patterns, a new normalization of measurable two-dimensional intensity patterns is employed, thereby enabling one to enhance the sensitivity of judgment to high-angle 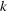 values, and eventually to improve attainable space resolution.

A two-dimensional intensity pattern depends on the direction of the incident X-ray beam with respect to the molecule-fixed coordinate system and an angle of rotation of the detector plane placed perpendicularly to the beam axis. When an angle 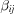 between the directions of the incident beam for a pair of two-dimensional intensity patterns is small, a high correlation line is observed in the two-dimensional correlation pattern as a straight line extending radially from the centre. The angle of the line in the correlation pattern gives a relative angle of rotation of the detector plane. The intensity of a high correlation line is unity near 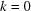 and becomes weaker at higher angles. The intensity reduces faster for larger values of 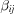.

The background of correlation patterns other than the high correlation line is characterized as a pattern of random appearance reflecting the irregular three-dimensional structures of biopolymers at the atomic level superimposed with the quantum noise. Owing to the deliberately adopted, new normalization of measurable two-dimensional intensity patterns, the mean value of the distribution in the background of two-dimensional correlation patterns turns out to vanish. The standard deviation 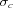 of the distribution around its vanishing mean becomes globally, but not monotonically, larger as 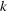 becomes larger. When the standard deviation 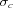 becomes larger than the intensity of a high correlation line, the latter becomes no longer recognizable. The recognizable length of a high correlation line becomes longer as the value of 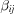 becomes smaller. The latter can be determined from the former.

When the value of 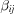 is smaller than a certain value, say, 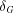 (therefore, when the recognizable length of the corresponding high correlation line becomes longer than a certain value, say, 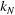), we classify the pair into the same group. To attain the best resolution, we should employ the largest possible value for 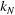. Operationally we determine the value of 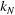, *the limiting k value* for correlation recognition, as the lower bound of the noise-dominant 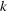 region in two-dimensional correlation patterns. Such a value 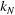 can be characterized theoretically as the value of 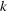 at which the standard deviation 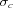 of the background distribution is 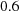. An analytic expression, equation (13), for the standard deviation is derived which is approximately a function of the wavenumber normalized by the Shannon length of the target molecule, *i.e.* 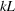, and an expected photon count 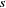 by a pixel at the position of the wavenumber 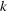. The quantity 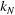 plays a central role in the method of analysis developed in this paper. It is shown that the structural resolution 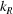 attainable by this method is given by 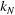.

In the method of identification of a high correlation line, upon which judgment of similarity of a pair of two-dimensional intensity patterns is based, effective information is extracted from the very noisy data in the range of wavenumbers up to the limiting value 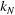 where the value of the standard deviation 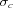 is 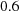. From the analytic expression for 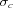, we can derive *the limiting photon count* 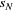, an expected photon count at *a limiting pixel*, approximately as a function of *the normalized resolution* 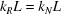 (Fig. 5 ▶). For a method of structure determination to be useful, the value of the normalized resolution should be in the range of 20–100. The corresponding value of the limiting photon count 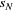 turns out to be in the range of 0.25–0.08. The proposed method of analysis is sufficiently sensitive to enable one to extract information from such low-photon-count noisy data. This high sensitivity has been attained by employing a new correlation function. When the molecular length 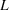 and the radial diffraction intensity density function 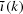 are known, the above relation between the normalized resolution 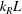 and the limiting photon count 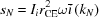 can be transformed to a relation giving the intensity 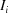 of the incident beam to be used to attain a resolution 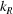.

The angle 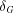 to define a range of classification groups is given by an inverse of the normalized limiting wavenumber, 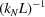. As a result, the number of classification groups 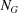 is given by 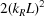.

A method of identifying common circles between an arbitrary pair of noise-reduced two-dimensional patterns is developed. For an arbitrary Ewald sphere, there exists a conjugate Ewald sphere which is centrosymmetric with respect to the origin of the wavenumber space. In the proposed method, when a common circle between two-dimensional patterns 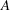 and 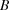 is searched, another common circle is searched at the same time between two-dimensional patterns 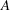 and 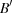, where 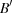 is a two-dimensional pattern on an Ewald sphere conjugate to the one on which 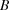 exists. The average number of two-dimensional patterns 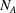 to be averaged in each group for identification of common circles has been shown to be given in terms of the limiting photon count 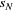 by 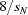.

The obtained theoretical expressions are used to evaluate values of important parameters for the two sample ‘molecules’ by assuming, respectively, two typical intensities of the incident beam. The results are shown in Table 1 ▶. We should note the very low limiting photon counts, highlighting the strength of the method developed here. We should also note that the predicted attainable resolutions are remarkably high. This is partly due to the strength of the method of analysis developed here, but also due to the assumed high intensities of the incident beam. The assumed values of intensity in Fig. 7 ▶ and Table 1 ▶ are in the range of around 10^21^ photons pulse^−1^ mm^−2^, which is far larger than the peak value of 1.6 × 10^16^ photons pulse^−1^ mm^−2^ reported in the recent experiment (Seibert *et al.*, 2011 ▶) carried out at the Linac Coherent Light Source (LCLS). Because the X-ray beam diameter reported in the experiment at LCLS is about 10 µm and a new technology (Mimura *et al.*, 2010 ▶) is now available to focus it down to 10 nm, the values assumed in this paper appear realistic. Since we developed the analysis in this paper under a tentative assumption that damage processes can be neglected, the indicated intensity is the lower bound to attain a targeted resolution. We are now carrying out a study of the damage processes to assess the upper bound of employable intensity. The number of two-dimensional patterns to be measured in Table 1 ▶ is not small, but appears tractable for real experiments. At the same time we should note that the number of classification calculations is not small.

## Figures and Tables

**Figure 9:**
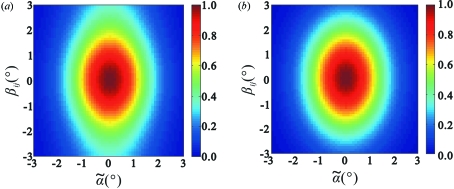
The quantity of equation (67) shown as a function of 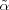 and 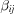 by assuming 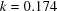
 Å^−1^ [value corresponding to 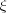 = 10° and 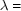 1 Å in equation (2)] and 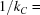 200 Å (the value for the HslUV complex). (*a*) Exact but numerically calculated value. (*b*) Value according to the approximate but analytic expression of equation (74).

**Table 1:** Resolution and necessary number of patterns and classification calculations expected for two sample ‘molecules’

	Lysozyme		HslUV complex	
Correlation length of intensity data 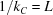	60 Å		200 Å	
Assumed intensity of incident beam 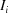 (photons pulse^−1^ mm^−2^)	5 × 10^21^	10^22^	5 × 10^20^	10^21^
Noise level becomes high at 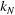 (Å^−1^)	0.48	0.99	0.28	0.55
Photon number 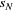 at 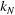	0.19	0.12	0.13	0.08
Resolution 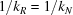 (Å)	2.08	1.01	3.57	1.82
Range of classification group 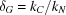 (°)	1.99	0.96	1.02	0.52
Number of classification group 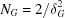	1.7 × 10^3^	7.1 × 10^3^	6.3 × 10^3^	2.5 × 10^4^
Number of two-dimensional patterns to be averaged 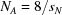	41	63	61	100
Necessary number of two-dimensional patterns 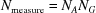	6.7 × 10^4^	4.5 × 10^5^	3.8 × 10^5^	2.4 × 10^6^
Number of classification calculations 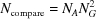	1.1 × 10^8^	3.2 × 10^9^	2.4 × 10^9^	5.8 × 10^10^

## Appendix A Relations between molecular orientation and the Ewald sphere

We define two right-handed coordinate systems, one fixed to the molecule and the other fixed to the experimental detector. They are defined, respectively, in terms of a set of mutually orthogonal unit vectors  fixed to the molecule and in terms of another set of mutually orthogonal unit vectors  fixed to the detector. The position of any point in the molecule can be expressed either in terms of coordinates  (here the superscript *t* means transpose) in the molecule-fixed coordinate system (MFCS) as  or in terms of coordinates  in the detector-fixed coordinate system (DFCS) as . Thus

The relative orientation between the MFCS and DFCS can be described in terms of a 3 × 3 orthogonal matrix  as

This is a shorthand notation for

where  is an element of the matrix . This is an orthogonal matrix, *i.e.* . By introducing equation (24) into equation (23), we have the following relation between coordinates in the two coordinate systems:

It is convenient to express the orthogonal matrix in terms of a Eulerian angle  as

where

The range of variation of the Eulerian angle is taken as follows:

Molecular structure is described by its electron density:

When the molecule is in the orientation described by an orthogonal matrix  with respect to the detector, the electron density in the DFCS is given by

The structure factor is calculated in the MFCS as

The structure factor in the DFCS is given by

This equation has the same structure as equation (33), indicating that the electron density and the structure factor behave in the same way for rotation. It follows from this that the wavenumber vectors  in the MFCS and  in the DFCS are related to each other similarly as in equation (26):

Experimentally, the diffracted X-rays from a single molecule in a certain specific orientation are measured by a two-dimensional detector as a continuous diffraction pattern. This diffraction pattern is given by the squared modulus of the structure factor on the following Ewald sphere in the wavenumber space in the DFCS,

Here  is the wavelength of X-rays used in the experiment. The incident X-rays are assumed to proceed to the negative direction of the third axis  of the DFCS. The first and second axes are taken on the surface of the detector. Equation (37) describes a sphere in the  space. The surface of the sphere contains the origin of the wavenumber space and its centre is located at .

We now introduce a polar coordinate  on the surface of this Ewald sphere by

The origin  of the polar coordinate corresponds to the origin  of the wavenumber space. This polar coordinate system is adopted so that when the detector is viewed from the direction of the incident X-rays, the detector’s positive horizontal and vertical axes coincide with the directions of  and , respectively. The observable diffraction pattern is given by equation (1) as

Because the orthogonal matrix  can be specified by a Eulerian angle , we sometimes write

We also identify various Ewald spheres by their corresponding orthogonal matrix .

Let us now locate the Ewald sphere in the MFCS. It is given by

The last equality means

This equation means that the Ewald spheres corresponding to  and  are essentially the same spheres giving the same surface. The former is obtained from the latter by rotating the latter anticlockwise around its third axis by angle .

Here, the different ways in which a Eulerian angle  appears in the two different coordinate systems may be worth noting. In the DFCS, a set of three angles describes the spatial orientation of the molecule. In the MFCS, the angles  are nothing but the polar coordinates of the incident X-ray beam axis  and the angle  describes the angle of rotation of the detector around this beam axis. Because of these clear meanings of the Eulerian angles in the MFCS, we employ them to describe molecular orientations rather than often mathematically better behaved quaternions.

Equation (42) suggests an experimental procedure of classifying various observable diffraction patterns  into groups only with respect to values of  and . Those with the same values of  and  but with different values of  are to be classified into the same group.

Because the electron density  is a real function, the structure factor satisfies the relation

Because of this relation, the same diffraction pattern is observed on a pair of different Ewald spheres. From equations (40), (39), (35) and (43) we have

The last equation indicates that two Ewald spheres, characterized by rotation matrices  and , respectively, exist always in a pair. They occupy positions centrosymmetric to each other with respect to the origin of the wavenumber space, and they are in touch with each other at the origin. When we introduce a right-handed coordinate system to the Ewald sphere  with its origin at the centre of the sphere, the corresponding coordinate system of the centrosymmetric Ewald sphere  is now left handed. This fact is understood as the two Ewald spheres having different handedness or parity.

## Appendix B Finding the relative orientation between a pair of unknown orientations from their corresponding diffraction intensity patterns

Real, experimentally observable diffraction patterns are subject to severe quantum noise. To cope with such noise, the same patterns should be measured many times and their means should be calculated to reduce the noise. In the following, the quantity of equation (39) is used under the assumption that such averaging has already been done so that the effect of noise can be neglected.

Let the two unknown orientations be given by

The corresponding Ewald spheres  and  are given, respectively, by

and

Because all these Ewald spheres have the same radii and their surfaces contain the origin  of the wavenumber space, they either contact at the origin or have a circular intersection, which also contains the origin.

Let us now study the intersecting circle between Ewald spheres  and . Let  on the first Ewald sphere  and  on the second Ewald sphere  be the same point on the intersecting circle,

Therefore, by setting

we have

These equations mean that the polar coordinates  on Ewald sphere  for points on the intersecting circle with Ewald sphere  are also polar coordinates on Ewald sphere  for points on the intersecting circle with Ewald sphere . Similarly, the polar coordinates  on Ewald sphere  for points on the intersecting circle with Ewald sphere  are also polar coordinates on Ewald sphere  for points on the intersecting circle with Ewald sphere .

We now calculate the intersecting circle between Ewald spheres  and , and that between Ewald spheres  and . We assume that  is given by equation (27). The centres of the three Ewald spheres, ,  and , are given in the MFCS by

Therefore

Let the centre of the intersecting circle between Ewald spheres  and  on Ewald sphere  be . Similarly, let the centre of the intersecting circle between Ewald spheres  and  on Ewald sphere  be . They are given by

We now proceed to describe a procedure to find the intersecting circles for a given pair of experimental diffraction patterns  and . When we move clockwise along the intersecting circle on the Ewald sphere , it appears as an anticlockwise motion along the intersecting circle on the Ewald sphere . Therefore, we calculate the following quantity for all assumed values of  in the range of :

This quantity should vanish on a certain circle for a certain value of . Because the intersecting circle contains the polar origin , it can be uniquely specified by the polar coordinate of its centre. When the quantity of equation (54) vanishes on a circle with its centre at , the polar coordinates of the centres of intersecting circles are  on  and  on . These polar coordinates of the centres are to be compared with equation (53). Therefore,

From these relations we have a half of equation (21) in the main text.

Let us now proceed to study the intersecting circle between Ewald spheres  and . By following similar procedures as above we have the following relations from equation (47):

By introducing similar quantities and following similar procedures, we have the following results:

To find the intersecting circle between Ewald spheres  and  for a given pair of experimental diffraction patterns  and , we calculate the following quantity for all assumed values of  in the range of :

When we move clockwise along the intersecting circle on Ewald sphere , it appears as an anticlockwise motion along the intersecting circle on Ewald sphere . Because Ewald spheres  and  have opposite parities, this motion appears as a clockwise motion along its centrosymmetric circle on Ewald sphere . This is the reason why the sign in front of  in  in equation (58), unlike in equation (54), is now plus. When the quantity of equation (58) vanishes on a circle with its centre at , the polar coordinates of the centres of intersecting circles are  on  and  on . These polar coordinates are to be compared with equation (57). Therefore

From these relations we have another half of equation (21) in the main text.

## Appendix C Derivation of equations (11), (12), (13) and (22)

The mean of the quantities of equations (6) and (7) with respect to the Poisson distribution is given by

When the quantity of equation (60) is averaged over the structure irregularity distribution, we have from equation (9) and the relation  due to the exponential distribution

where  is an angle between the two  vectors given explicitly as follows according to equation (41):

When the two  vectors are always significantly different, equation (62) reduces to

The average of the quantity of equation (61) over the structure irregularity distribution is given by

By introducing an approximation

we see from equations (64) and (65) that  vanishes as mentioned in the main text. Also, from equations (62), (65) and (66) we have

This is an expected expression when all background features having random appearance due to the quantum noise and the structure irregularity distribution are erased from such high correlation lines as observed in Figs. 2 ▶(*a*) and 2 ▶(*b*).

We now proceed to simplify equation (67). At first we calculate , an angle between the two vectors of equation (63). The magnitude of the two vectors is given by equation (2). To calculate the inner product between the two vectors, we introduce the relative Eulerian angle  by

Here  is the angle between beam directions given by equation (5). In terms of the relative Eulerian angle, we have

The quantity of equation (67) is to be obtained by substituting  from this equation. Let us write the result of the integration with respect to  in equation (67) by writing three independent variables explicitly as . Here, remember that  and  are related by equation (2). Even though it is difficult to derive an analytic form of this quantity, we can calculate it numerically. Fig. 9 ▶(*a*) shows an example of this quantity obtained numerically. For practical applications we want to have an analytic form of this quantity even when it may be somewhat approximate. To derive an approximate but analytic form we at first notice that the integrand of equation (67) has a significant contribution only when  and  are small. By using this fact we Taylor-expand both sides of equation (69) in terms of ,  and , and retain only up to the second-order terms to obtain

The quantity of equation (67), obtained numerically by using this expression, was found to have almost no difference from , meaning that equation (71) is a very good approximation to equation (69). Even for this expression of equation (71), the derivation of an analytic form for the integral of equation (67) is difficult. In this situation we introduce a drastic approximation of replacing the quantity of equation (71) by its mean

and use the value of  thus obtained in equation (67). Writing the result thus obtained as  we have

For practical applications we can approximate this expression further as

Fig. 9 ▶(*b*) is a plot of this quantity. By comparing it with Fig. 9 ▶(*a*), we see that this simple analytic form is a reasonably good approximation.

Equation (74) is equation (11) of the main text. Equation (12) can be obtained from equations (68) and (70) by assuming that  and therefore both  and  are small quantities of the same order.

For the purpose of deriving equation (13), we evaluate the standard deviations of the quantities appearing in equations (6) and (7). After some calculations they are obtained as

During the derivation of this equation we assumed that the values of the diffraction intensity density at the neighbouring Shannon pixels on a circle are not correlated. This means that the distance in the wavenumber space between the neighbouring Shannon pixels must be larger than the correlation length  of equation (10). Because the correlation fades quickly beyond , we assume that equation (75) still holds for this distance. Therefore we have equation (15). The standard deviation of the correlation pattern is then given by

from which we have equation (13).

We now proceed to derive equation (22). We study how the value of the product  is determined for common circles to be identified. At first we note that a sum of Poisson distributions is a Poisson distribution. Therefore, a sum of  two-dimensional patterns is equal to one sample of a two-dimensional pattern whose expected photon number is given by , which we shall write as  in equation (77) below. We shall also write the mean of  on a sphere  as . Now we consider the distribution of values of the difference  (1) on and (2) off the common circle. (1) On the common circle,  is given by a difference of two integers, both given by a Poisson distribution. Its mean vanishes of course. The mean of its absolute value may be estimated approximately as

(2) Off the common circle, the values of  and  are both distributed according to the two distributions, the Poisson distribution and the exponential distribution. The composite distribution is given by

For this distribution, the mean of the absolute value is estimated approximately as follows:

Let us assume that, for clear recognition of  out of , the following condition must be satisfied up to : , which means

Equation (22) follows directly from this equation.
